# New records and genetic diversity of *Mycoplasma ovis* in free-ranging deer in Brazil

**DOI:** 10.1017/S0950268819002218

**Published:** 2020-01-14

**Authors:** Marcos Rogério André, José Maurício Barbanti Duarte, Luiz Ricardo Gonçalves, Ana Beatriz Vieira Sacchi, Márcia Mariza Gomes Jusi, Rosangela Zacarias Machado

**Affiliations:** 1 Laboratório de Imunoparasitologia, Departamento de Patologia Veterinária, Universidade Estadual Paulista (Unesp), Faculdade de Ciências Agrárias e Veterinárias, Jaboticabal, SP, Brasil; 2 Núcleo de Pesquisa e Conservação de Cervídeos, Departamento de Zootecnia, Universidade Estadual Paulista (Unesp), Faculdade de Ciências Agrárias e Veterinárias, Jaboticabal, SP, Brasil

**Keywords:** 16S rRNA genotypes, haemoplasmas, *Mazama bororo*, *Mazama gouazoubira*

## Abstract

Cervids represent a mammal group which plays an important role in the maintenance of ecological balance. Recent studies have highlighted the role of these species as reservoirs for several arthropods-borne pathogens. Globally, hemotropic mycoplasmas (haemoplasmas) are emerging or remerging bacteria that attach to red blood cells of several mammals species causing hemolytic anaemia. Therefore, the aim of this study was to investigate the occurrence and assess the phylogenetic positioning of *Mycoplasma ovis* in free-ranging deer from Brazil. Using a polymerase chain reaction targeting the 16S rRNA region, 18 (40%) out of 45 sampled deer were positive to *M. ovis.* Among the nine sequences analysed, four distinct genotypes were identified. The sequences detected in the present study were closely related to sequences previously identified in deer from Brazil and the USA. On the other hand, the Neighbour-Net network analysis showed that the human-associated *M. ovis* genotypes were related to genotypes detected in sheep and goats. The present study shows, for the first time, the occurrence of *M. ovis* in *Mazama gouazoubira* and *Mazama bororo* deer species, expanding the knowledge on the hosts harbouring this haemoplasma species. Once several deer species have your population in decline, additional studies are needed to evaluate the pathogenicity of *M. ovis* among deer populations around the world and assess its potential as reservoir hosts to human infections.

## Introduction

Cervids represent a diverse mammal group, containing more than 60 species described [1], playing an important role in the maintenance of ecological balance [2]. Among the eight cervids species occurring in Brazil, *Odocoileus virginianus* (white-tailed deer), *Ozotocerus bezoarticus* (pampas deer), *Blastocerus dichotomus* (marsh deer), *Mazama nemorivaga (*Amazonian brown brocket), *Mazama gouazoubira* (gray brocket deer), *Mazama nana* (Brazilian dwarf brocket), *Mazama americana* (Red brocket) and *Mazama bororo* (small red brocket deer) [3–6], *B. dichotomus, M. bororo* and *M. nana* species are classified as vulnerable (IUCN: accessed in 2019 May). Specifically, the *B. dichotomus* and *M. gouazoubira* are included in the Brazilian National Action Plan for Conservation of Endangered South America Deer and have your population in decline, mainly because of habitat destruction and hunting (https://www.iucnredlist.org).

Hemotropic mycoplasmas (haemoplasmas) belong to Mollicutes Class and Mycoplasmataceae Family [7]. These agents are epierythrocytic bacteria that attach to red blood cells from a wide variety of mammals, including humans [8–14]. Although haemoplasma infection generally shows chronic and subclinical courses, affected mammals can develop hemolytic anaemia, mainly when immunosuppressed [15–17].

*Mycoplasma ovis*, a zoonotic pathogen frequently detected in sheep and goats [18 19], have been detected in *O. virginianus* [20, 21] and *Rangifer tarandus* species [22] kept in captivity in the USA and in free-ranging *Cervus nippon* species in Japan [23]. In Brazil, *M. ovis* has already been detected in free-ranging *B. dichotomus* and *O. bezoarticus* from three distinct Brazilian areas, namely Pantanal (state of Mato Grosso do Sul, Midwestern Brazil), Emas National Park (state of Goiás state, Midwestern Brazil) and Paraná river basin (São Paulo state, southeastern Brazil) [24]. Additionally, *M. ovis* DNA was detected in blood samples from *M. nana*, *M. americana* and *B. dichotomus* species maintained in captivity at Bela Vista Biological Sanctuary (Paraná state, Southern Brazil) [25].

The present study aimed to investigate the occurrence and assess the genetic diversity of *M. ovis* in free-ranging deer sampled in four Brazilian states.

## Materials and methods

### Ethical statement

Deer blood sampling was conducted by Professor José Maurício Barbanti Duarte (IBAMA Registration number 263703), from the Department of Animal Sciences, FCAV – UNESP, Jaboticabal, with license number 10636-1 provided by IBAMA, between 1996 and 2011.

### Number and origin of sampled deer

In total 34 and 11 DNA samples were extracted from *Mazama* spp. and *Ozotoceros bezoarticus* buffy coats samples, respectively. Among these animals, 21 *M. gouazoubira* and 11 *O. bezoarticus* were captured in the Pantanal Sul Matogrossense (MS); 4 *M. gouazoubira* in the region of the Serra da Mesa Hydroelectric Power Plant (GO); 4 *M. bororo* and 2 *M. gouazoubira* in the Intervales State Park (SP); and 3 *M. americana* in the Iguaçu National Park (PR) [26].

### DNA extraction

DNA was extracted from 200 µl of each buffy coat samples using the DNeasy Blood and Tissue kit (Qiagen, Valencia, CA, USA), according to the manufacturer's instructions. The DNA concentration and absorbance ratio (260/280 nm) were measured using a Nanodrop spectrophotometer (Thermo Scientific, Waltham, MA, USA).

### Molecular detection of *M. ovis*

A previously described conventional (c) polymerase chain reaction (PCR) protocol based on the fragment (~1300 bp) of the 16S rRNA gene was used for detection of *M. ovis* DNA [24]. Briefly, 5 µl of DNA was used as a template in 25 µl reaction mixtures containing 10X PCR buffer, 1.0 mM MgCl_2_, 0.6 mM deoxynucleotide triphosphate (dNTPs) mixture, 1.5U of *Taq*DNA polymerase (Life Technologies) and 0.5 µM 16S-Fw 5′-ATGCAAGTCGAACGAGTAGA-3′, and 16S-Rv 5′- TGATACTTTCTTTCATAGTTTG-3′ primers. PCR amplifications were performed at 94°C for 5 min followed by 39 repetitive cycles of 94°C for 1 min, 51.6°C for 30 s and 72°C for 1 min, followed by a final extension at 72°C for 5 min. *Mycoplasma ovis* DNA obtained from a naturally infected Brazilian marsh deer [25] and ultra-pure sterile water were used as positive and negative controls, respectively.

### Sequencing and analyses of sequences

Randomly selected PCR products were purified using Silica Bead DNA Gel Extraction Kit (Fermentas, São Paulo, SP, Brazil). Purified amplified DNA fragments from positive samples were submitted to sequence confirmation in an automatic sequencer (ABI Prism 310 Genetic Analyser – Applied Byosystem/Perkin Elmer) in both directions. Lastly, in order to correctly determine the nucleotide composition, the electropherograms were submitted to PhredPhrap program [27]. The Phred quality score (peaks around each base call) was established higher than 20 (99% in accuracy of the base call). Subsequently, the sequences were submitted to phylogenetic analyses. The sequences amplified in the present study were deposited in GenBank data base under accession numbers: (MK919446-MK919454).

### Phylogenetic analyses

Haemoplasmas-16S rRNA sequences were identified by BLASTn using the Megablast (following default parameters), aligned with sequences available in GenBank using Clustal/W [28] and adjusted in Bioedit v. 7.0.5.3 [29]. The phylogenetic analysis was performed using Maximum Likelihood (ML) method. The ML phylogenetic analysis was inferred with RAxML-HPC BlackBox 7.6.3 [30]. The analysis (ML) was performed in CIPRES Science Gateway [31]. The Akaike Information Criterion (AIC) available on MEGA software was applied to identify the most appropriate model of nucleotide substitution. GTR + G + I model was chosen as the most appropriate for the phylogenetic analysis of the 16S rDNA alignment.

### Identification and genetic relationship of *M. ovis* genotypes

The 16S rRNA aligned sequences amplified in the present study were utilised to identify the number of genotypes, calculate the nucleotide diversity (*π*), the polymorphic level (genotype diversity – [Gd]) and the average number of nucleotide differences (*K*) using the DnaSP v5.10 [32]. To investigate the genetic relationship among *M. ovis* genotypes detected in deer in the present study and those previously detected in sheep, goats and humans and retrieved from GenBank, a Neighbour-Net network was constructed using the pairwise genetic distances with SplitsTree v4.10 [33]. Additionally, the different genotypes identified were submitted to TCS network inferred using the Population Analysis with Reticulate Trees (popART) (v. 1.7) [34].

## Results

### Occurrence of *M. ovis* and BLASTn analysis

A total of 18 (40%) out of 45 sampled deer were positive to *M. ovis*, including 11 brown brocket deer (*M. gouazobira*) (10 from Mato Grosso do Sul state and one from Goiás state), one small red brocket deer (*M. bororo*) from São Paulo state, one red brocket deer (*M. americana*) from Paraná state and five pampas deer (*O. bezoarticus*) from Mato Grosso do Sul state. The BLASTn analysis performed on nine positive samples randomly selected showed that all sequences amplified in the present study shared 99% identicalness with *M. ovis* previously detected in deer from Brazil (HQ197746, HQ634377 and HQ634378) and USA (FJ824847). All sequences amplified in the present study showed query coverage of 100%.

### Phylogenetic and genotype analyses

**Fig. 1.**
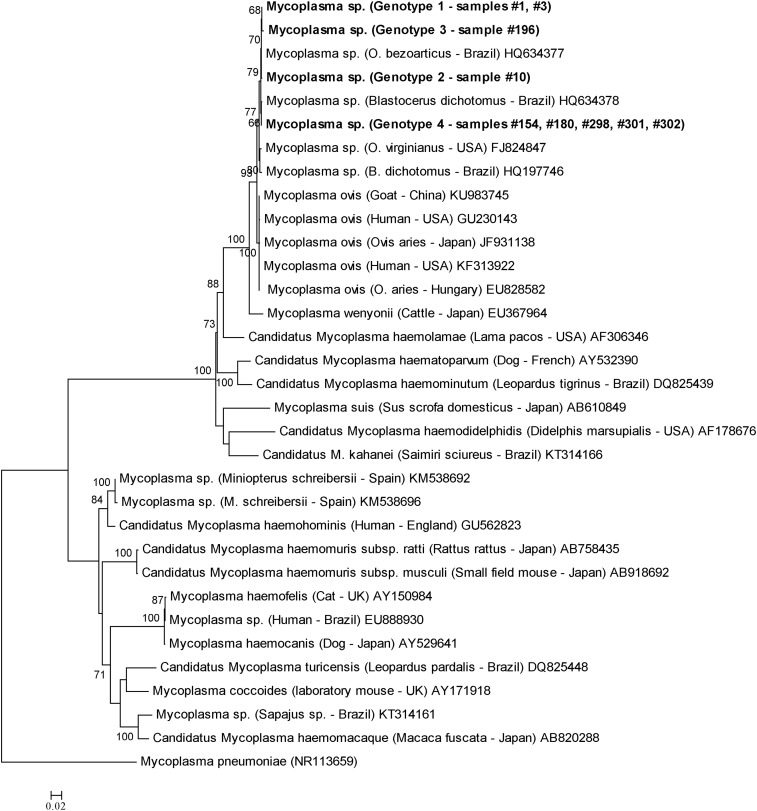
Phylogenetic relationships within the *Mycoplasma* genus based on 16S rRNA gene (1056 bp). The tree was inferred by using the Maximum Likelihood (ML) with the GTR + G + I model. The sequences detected in the present study are highlighted in bold. The numbers at the nodes correspond to bootstrap values higher than 60% accessed with 1000 replicates. *Mycoplasma pneumoniae* (NR113659) was used as outgroup.

**Fig. 2.**
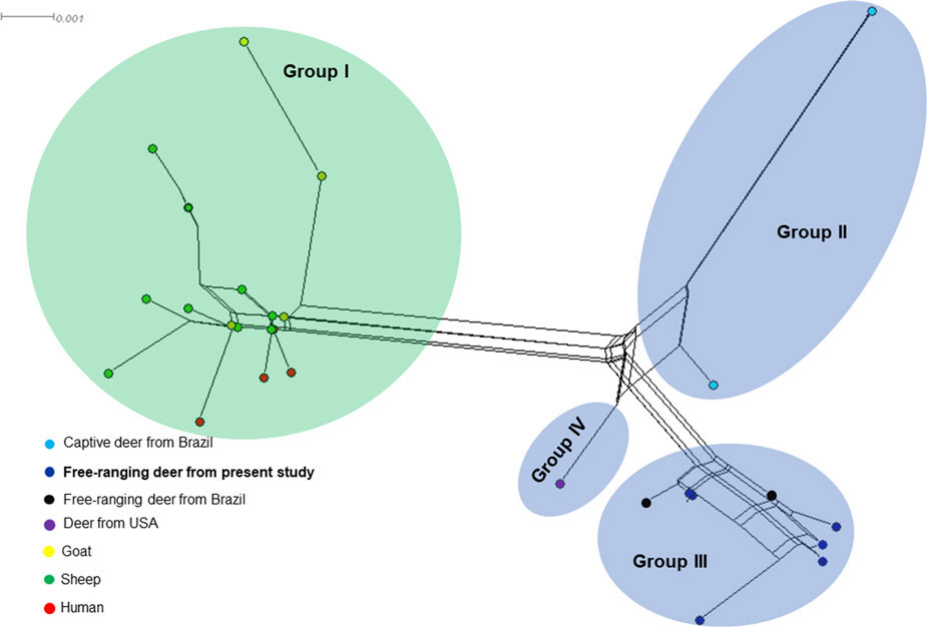
Neighbour-Net network inferred using 16S *Mycoplasma ovis* genotypes detected in different hosts. The goat, sheep and human genotypes were grouped in Group I. Groups II, III and IV refer to deer genotypes.

**Fig. 3.**
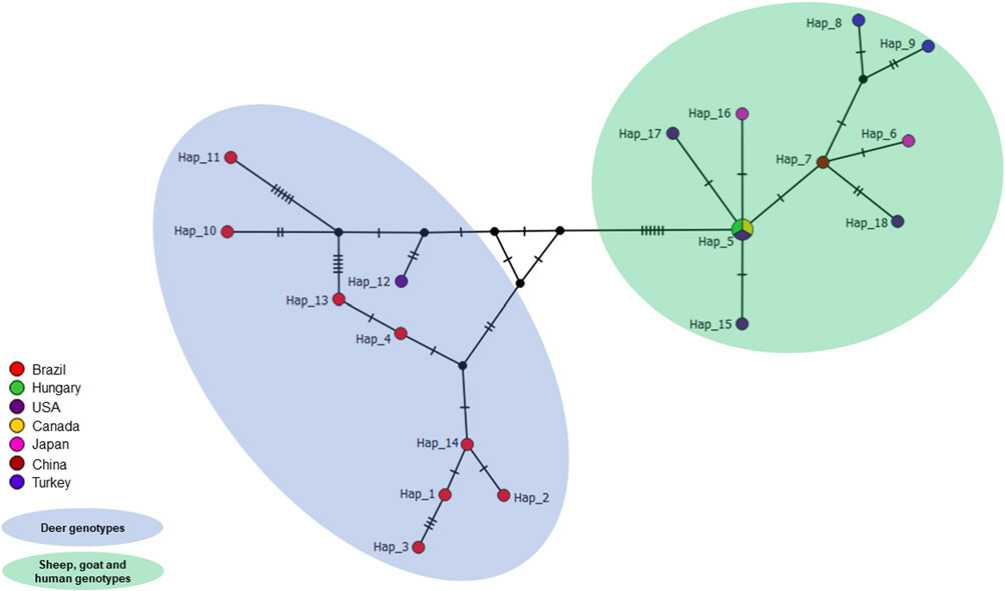
TCS network inferred using the 16S *Mycoplasma ovis* genotypes detected in different hosts. The goat, sheep and human genotypes are highlighted in light blue. The deer genotypes are highlighted in light green.

The amplified sequences were positioned within the *M. suis* group and clustered with others *M. ovis* sequences detected in animals and humans. The sequences detected in the present study were closely related to sequences previously identified in deer from Brazil and the USA, albeit lightly apart from *M. ovis* genotypes detected in sheep, goats and humans. The ML analysis was supported by high bootstrap values (Fig. 1). In agreement with ML analysis, but with a marked separation among *M. ovis* sequences identified in sheep, goats, humans and those amplified in deer, the network analysis showed that the *M. ovis* sequences were divided into four groups. All analysed *M. ovis* 16S rRNA sequences detected in sheep, goats and humans were clustered into group I. In addition, the groups II, III and IV comprise the *M. ovis* sequences identified in deer (Fig. 2). Finally, the TCS network showed similar results, since all deer genotypes clustered together and separated from those genotypes detected in sheep, goats and human and identified in different countries (Fig. 3). Besides, the findings are supported by the divergence scores among the different genotypes (Table 1).

**Table 1.** Divergence scores among the different *M. ovis* genotypes identified in deer, goats, sheep and human. The genotypes were previously assessed with DnaSP v5.10. The pairwise distance matrix was estimated using the Mega 5.05

Genotypes	1	2	3	4	5	6	7	8	9	10	11	12	13	14	15	16	17	18
1	–																	
2	0.002	–																
3	0.003	0.005	–															
4	0.003	0.003	0.004	–														
5	0.013	0.013	0.015	0.010	–													
6	0.014	0.014	0.016	0.011	0.001	–												
7	0.013	0.013	0.015	0.010	0.000	0.001	–											
8	0.015	0.015	0.017	0.013	0.002	0.003	0.002	–										
9	0.016	0.016	0.018	0.014	0.003	0.004	0.003	0.003	–									
10	0.009	0.009	0.013	0.008	0.011	0.013	0.011	0.014	0.014	–								
11	0.014	0.014	0.017	0.013	0.016	0.017	0.016	0.018	0.017	0.008	–							
12	0.008	0.008	0.012	0.007	0.010	0.011	0.010	0.013	0.014	0.005	0.011	–						
13	0.004	0.004	0.005	0.001	0.012	0.013	0.012	0.014	0.015	0.007	0.012	0.008	–					
14	0.001	0.001	0.004	0.002	0.012	0.013	0.012	0.014	0.015	0.008	0.013	0.007	0.003	–				
15	0.014	0.014	0.016	0.012	0.001	0.002	0.001	0.003	0.004	0.013	0.017	0.011	0.013	0.013	–			
16	0.014	0.014	0.016	0.011	0.001	0.002	0.001	0.003	0.004	0.013	0.017	0.011	0.013	0.013	0.002	–		
17	0.014	0.014	0.016	0.012	0.001	0.002	0.001	0.003	0.004	0.013	0.017	0.011	0.013	0.013	0.002	0.002	–	
18	0.015	0.015	0.017	0.013	0.002	0.003	0.002	0.004	0.005	0.014	0.018	0.013	0.014	0.014	0.003	0.003	0.003	–

Among the nine sequences detected in the present study, four distinct genotypes were identified (Table 2). The genotype diversity (*Gd*), nucleotide diversity (per site = *π*) and the average number of nucleotide differences (*K*), were 0.694, 0.002 and 2.22, respectively.

**Table 2.** Host, sampling sites and identification of the identified genotypes

Genotypes	Host	Sampling sites	Samples	GenBank accession numbers
Genotype 1	Pampas deer	Mato Grosso do Sul	#1	MK919446
			#3	MK919450
Genotype 2	Pampas deer	Mato Grosso do Sul	#10	MK919447
Genotype 3	Brown brocket deer	Goiás	#196	MK919448
Genotype 4	Brown brocket deer	Mato Grosso do Sul	#154	MK919449
			#180	MK919451
			#298	MK919452
			#301	MK919453
			#302	MK919454

## Discussion

Recent studies have suggested a possible co-evolution among haemoplasmas and their respective hosts [11, 21, 35]. However, little is known about the origin, dispersion and evolutionary aspects of hemotropic mycoplasmas [12].

Globally, haemoplasmas comprise emerging or re-emerging zoonotic pathogens that affect domestic and wild animals. Previously, based upon molecular diagnosis, *M. ovis* was detected in a veterinarian in Texas, USA, which was coinfected with *Bartonella henselae* [36]. More recently, *M. ovis* was detected in patients without and with extensive arthropods or animal contact [10]. Although the pathogenicity and reservoirs of *M. ovis* infecting humans are still unknown, the present study showed, for the first time, that the human-associated *M. ovis* genotypes were closely related to those detected in goat and sheep. However, further studies are needed in order to assess this issue as well as the transmission routes of this haemoplasma species.

The occurrence of *M. ovis* detected in the present study (40%) was lower than that previously detected among free-ranging (58%) and captive cervids (87%) from Brazil [24 25]. A high occurrence of *M. ovis* in deer could be related to the transmission routes involved. Thus, blood-sucking arthropods (ticks and flies), as well as vertical transmission, may play a role in the widespread infection of this haemoplasma species among deer [24].

Interesting, the phylogenetic relationship of the 16S rRNA sequences amplified in the present study showed that the *M. ovis* genotypes detected in Brazilian deer clustered together and were lightly distant from those detected in goats, sheep and humans, suggests a possible specificity among the different genotypes of *M. ovis* and their respective vertebrate hosts. In agreement to ML analysis, the networks also confirmed this result and showed clearly that *M. ovis* detected in goats, sheep and humans are genetic related each other, whereas *M. ovis* from deer could be classified as a distinct genogroup. However, more studies targeting different genes, analysing additional sequences and verifying other biological aspects are needed.

Although analysing few sequences, a low genetic diversity was observed in *M. ovis* sequences amplified from Brazilian deer. These results were expected since the 16S rRNA region show low genetic variation. However, it seems like different genotypes circulate in deer populations in the Pantanal region, state of Mato Grosso do Sul state. Two different genotypes were identified circulating on *O. bezoarticus* from the same region. On the other hand, only one genotype was detected in *M. gouazobira* individuals caught between 1996 and 2010, suggesting possible maintenance of this genotype over time among deer population in this region.

Among the eight Brazilian deer species, *M. ovis* has already been detected in four of them, namely *B. dichotomus*, *O. bezoarticus*, *M. nana* and *M. americana* [24 25]. Additionally, the present study shows, for the first time, the occurrence of *M. ovis* in *M. gouazoubira* and *M. bororo* species. The wide distribution and the number of deer species infected support the role of cervids in the maintenance of *M. ovis* transmission cycle in the environment. Once several deer species have your population in decline or are classified as vulnerable, additional studies are needed to evaluate the pathogenicity of *M. ovis* among deer populations from Brazil and around the world, as well as the environmental and biological factors which contribute to deer infection.

## Conclusion

The present study shows, for the first time, the occurrence of *M. ovis* in *M. gouazoubira* and *M. bororo* deer species, expanding the knowledge on the hosts harbouring this haemoplasma species. The *M. ovis* genotypes found in deer in Brazil clustered with other sequences previously detected in cervids, albeit slightly apart from humans, sheep and goats-associated genotypes, suggesting a probable specificity of *M. ovis* genotypes to groups of vertebrate hosts.
